# Regional thickness of the compacted and trabeculated layers of the left ventricle in young patients presenting with ventricular dysrhythmias, a Cardiac MRI study

**DOI:** 10.1186/1532-429X-14-S1-O106

**Published:** 2012-02-01

**Authors:** Moneal Shah, Lynette Duncanson, Ellen Cummings, Nathaniel Reichek, Jie J Cao

**Affiliations:** 1 Allegheny General Hospital, Pittsburgh, PA, USA; 2 St. Francis Hospital, Roslyn, NY, USA

## Summary

We sought to characterize the compacted and trabeculated layers of the left ventricle in patients presenting with ventricular dysrhythmias. Our results showed the dysrhythmic group had a statistically significant increase in the trabecular thickness as well as increased prevalence of trabecular to compacted thickness ratio.

## Background

Cardiac MRI (CMR) is increasingly being used clinically to assess patients with ventricular dysrhythmias. Structural heart disease such as non-compaction is associated with dysrhythmic risk. Recent literature has characterized the regional thickness of the compacted and trabeculated layers of the left ventricle (LV) in normal subjects but not in patients with ventricular dysrhythmias. We sought to characterize the regional thickness of the compacted and trabeculated layers in a young community-based ventricular dysrhythmia population.

## Methods

The study consisted of 98 patients under the age of 50 who had undergone CMR for ventricular dysrhythmia evaluation. These patients were compared to 26 age-matched normal subjects. Patients who had known cardiomyopathy or myocardial infarction and those referred for AICD evaluation were excluded. SSFP cine images were used to assess cardiac structure and function. Intravenous gadolinium was given for late gadolinium enhancement (LGE) assessment. Regional thickness of the compacted and trabeculated layers were assessed in short axis cine views in 16 of the standard 17 segments at end-diastole. The trabeculated layer thickness and the ratio of trabeculated to compacted layer were compared between the dysrhythmic and control group.

## Results

The mean age was 37 years and 56 (57%) were women. In the dysrhythmic group, 45 had premature ventricular contractions (PVC) and 55 had ventricular tachycardia (VT). Mean LV ejection fraction was 54±7% in the dysrhythmia group. LGE was present in 10 cases (10%) but none showed an infarct pattern. On average, the trabeculated layer was significantly larger in the dysrhythmic group than in control group: 3.7±1.4 mm vs. 3.0±1.0 mm at base (p<0.001), 4.6±1.3 mm vs. 3.4±1.0 mm in mid segment (p<0.001) and 5.4±1.5 mm vs. 3.5±0.9 mm in apical segment (p<0.001). The prevalence of trabeculated to compacted ratio ≥2.0 was 19% in controls, 20% in PVC group and 20% in VT group (p=NS). In contrast, the prevalence of a ratio ≥2.3 was 0 in controls, 16% in PVC group and 11% in VT group (p=0.01). The most common areas where the ratio exceeded 2.3 were mid-anterior, mid-anterolateral, apical anterior and apical lateral walls.

## Conclusions

In this young cohort with ventricular dysrhythmias, the trabeculated layer was significantly thicker than age-matched controls. Moreover, a trabeculated to compacted ratio greater than 2.3 was only present in patients with ventricular dysrhythmias, but not in normal controls. Our findings suggest that increased thickness of the trabeculated layer in young subjects may be associated with the risk of ventricular dysrhythmia.

## Funding

None.

